# Bilateral Congenital Cholesteatoma in a 13-Year-Old Boy

**DOI:** 10.7759/cureus.90298

**Published:** 2025-08-17

**Authors:** Yahya Boualam, Achraf Sbai, Drissia Benfadil, Azzedine Lachkar, Fahd El Ayoubi El Idrissi

**Affiliations:** 1 Department of Otolaryngology - Head and Neck Surgery, Faculty of Medicine and Pharmacy, Mohammed First University of Oujda, Oujda, MAR; 2 Department of Otolaryngology - Head and Neck Surgery, Mohammed VI University Hospital, Oujda, MAR

**Keywords:** bilateral congenital cholesteatoma, computed tomography (ct), ossicular chain lysis, ossiculoplasty, otologic surgery, otoscopy

## Abstract

A congenital cholesteatoma is a cystic lesion composed of keratinizing squamous epithelium that arises behind an intact and normal-appearing tympanic membrane. It typically appears as a white, pearl-like mass and often presents with progressive conductive hearing loss. Although congenital cholesteatoma itself is rare, the bilateral form is extremely uncommon. Diagnosis relies on clinical examination, particularly otoscopy, and imaging may support the evaluation. Treatment is exclusively surgical to prevent complications and preserve hearing. We report the case of a 13-year-old boy diagnosed with bilateral congenital cholesteatoma, successfully managed with staged surgical excision.

## Introduction

Congenital cholesteatoma is defined as a cystic mass of keratinizing epithelium located behind an intact tympanic membrane, without any history of tympanic membrane perforation, otorrhea, or previous otologic surgery [1,2]. It is a rare pathology, accounting for approximately 1% to 5% of all cholesteatomas in most published series [3]. The clinical presentation depends on the location and extent of the lesion. It may manifest as hearing loss, facial nerve paralysis, or even intracranial complications. The lesion typically progresses by expanding within the tympanic cavity and causing ossicular destruction, which highlights the importance of early diagnosis and intervention to prevent potentially serious complications [4].

We report a case of bilateral congenital cholesteatoma in a 13-year-old boy who presented with hearing loss noticed by his mother.

## Case presentation

**Figure 1:**
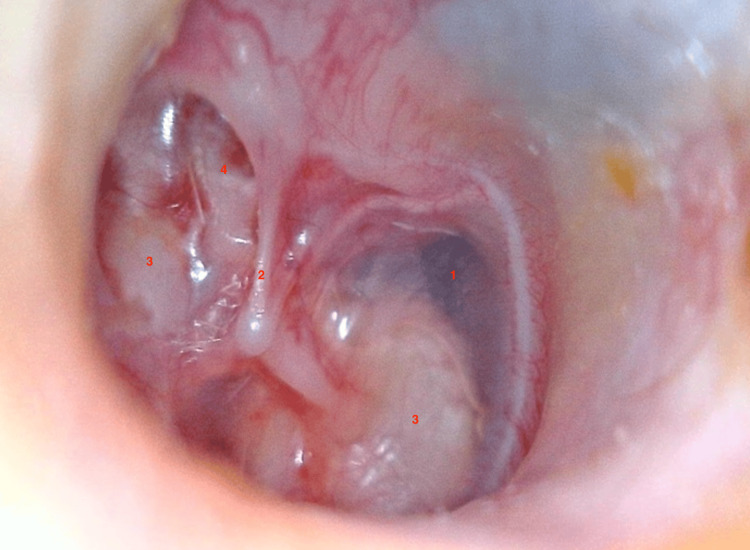
Left ear otoscopy: a whitish mass (3) with well-defined, convex borders occupying the anterosuperior, anteroinferior, and posteroinferior quadrants of the tympanic membrane. The mass is located behind an intact, non-perforated tympanic membrane (1), which appears retracted in the anterosuperior quadrant (4), and the handle of the malleus is prominent and clearly visible (2).

**Figure 2:**
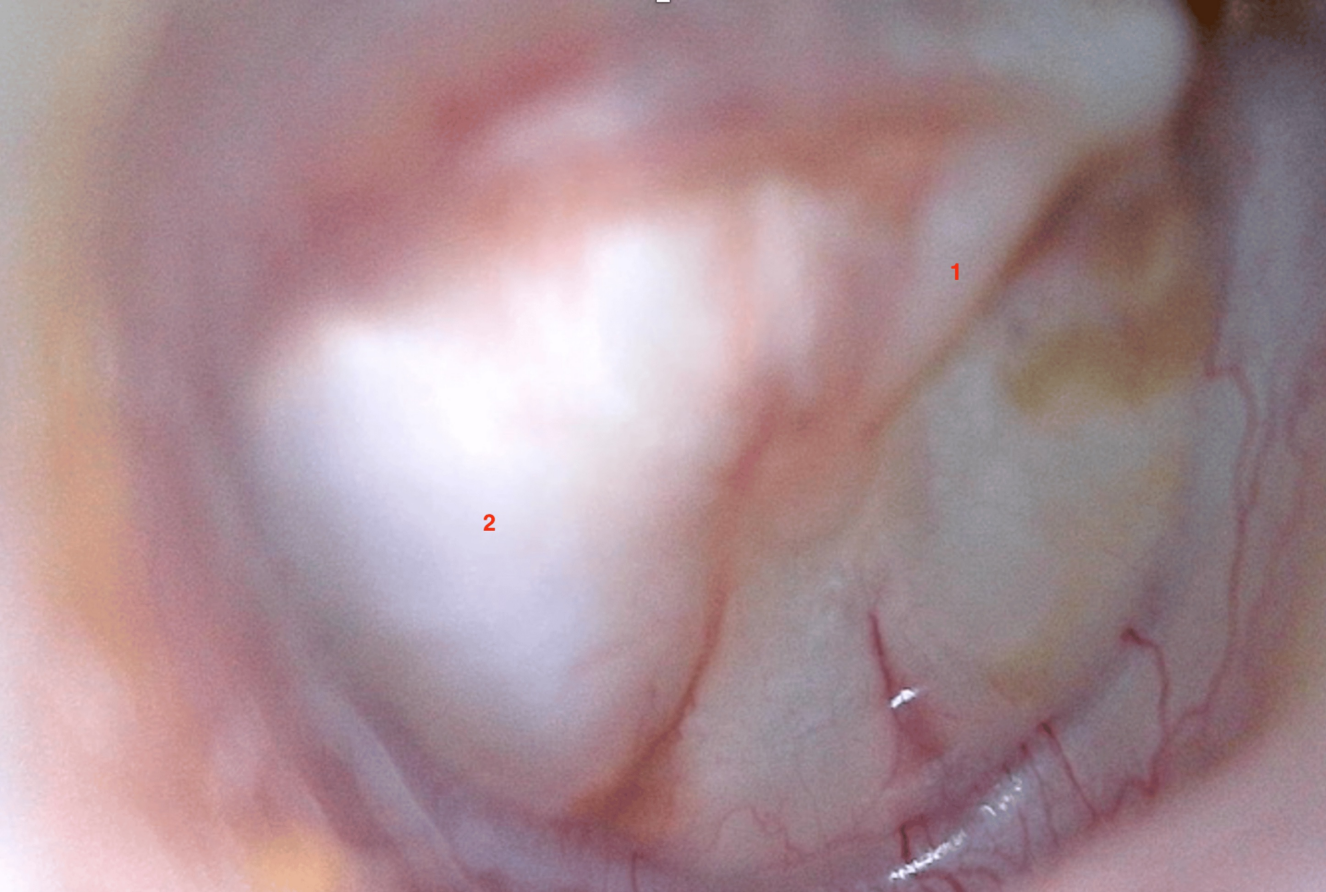
Right-ear otoscopy showing a large retrotympanic white mass causing outward convexity of the intact tympanic membrane (2) with a visible handle of the malleus (1).

A 13-year-old male patient presented with a three-year history of progressive hearing loss. There was no family history of hearing impairment, no episodes of otorrhea, and no prior otologic surgery. Otoscopic examination showed normal external auditory canals on both sides. However, retrotympanic whitish masses with a pearly appearance were observed (Figures 1, 2), more prominent on the right side (Figure 2), suggestive of congenital cholesteatoma.

**Figure 3:**
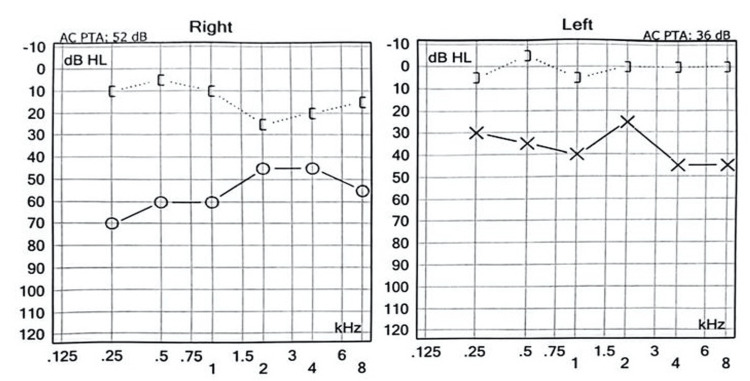
Pure-tone audiometry confirmed bilateral conductive hearing loss, with a more significant impairment in the right ear (average air conduction threshold: 67.5 dB HL) compared to the left ear (35 dB HL).

Pure-tone audiometry demonstrated bilateral conductive hearing loss, more pronounced on the right side (Figure 3), indicating middle ear dysfunction consistent with the underlying pathology.

**Figure 4:**
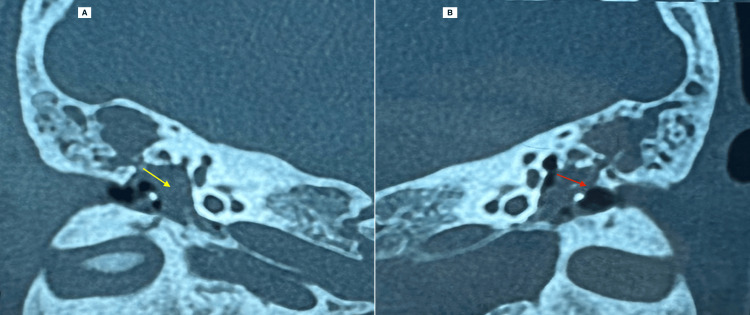
(A, B) Coronal sections of the temporal bone CT demonstrate bilateral complete hypodense opacification of the middle ears (yellow arrow) and mastoid antra, associated with ossicular chain lysis (red arrow) and erosive changes of the tegmen tympani and the lateral attic walls.

A high-resolution computed tomography (CT) scan of the temporal bones was performed (Figure 4), revealing radiological features consistent with bilateral chronic cholesteatomatous otitis media. The scan demonstrated complete opacification of the middle ear cavities (yellow arrow) and mastoid air cells on both sides, suggestive of soft tissue density compatible with cholesteatoma. In addition, there was clear evidence of bilateral ossicular chain erosion (red arrow), particularly involving the long process of the incus and stapes superstructure. These findings are indicative of an aggressive and expansive lesion, typical of chronic cholesteatomatous pathology, and help confirm the extent and bilaterality of the disease.

**Figure 5:**
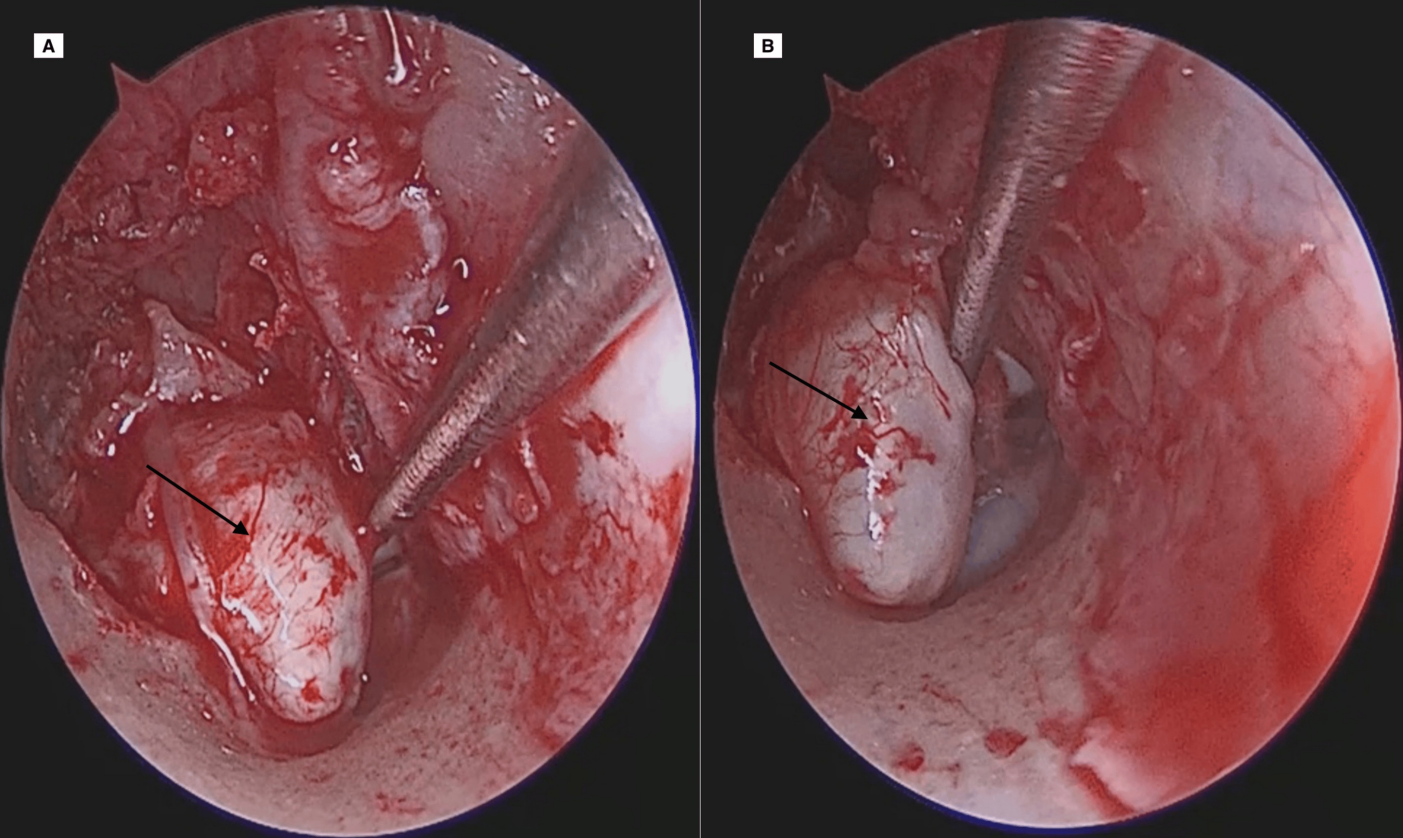
(A, B) Endoscopic view after elevation of the tympanomeatal flap showing the congenital cholesteatoma sac (black arrow) within the middle ear.

**Figure 6:**
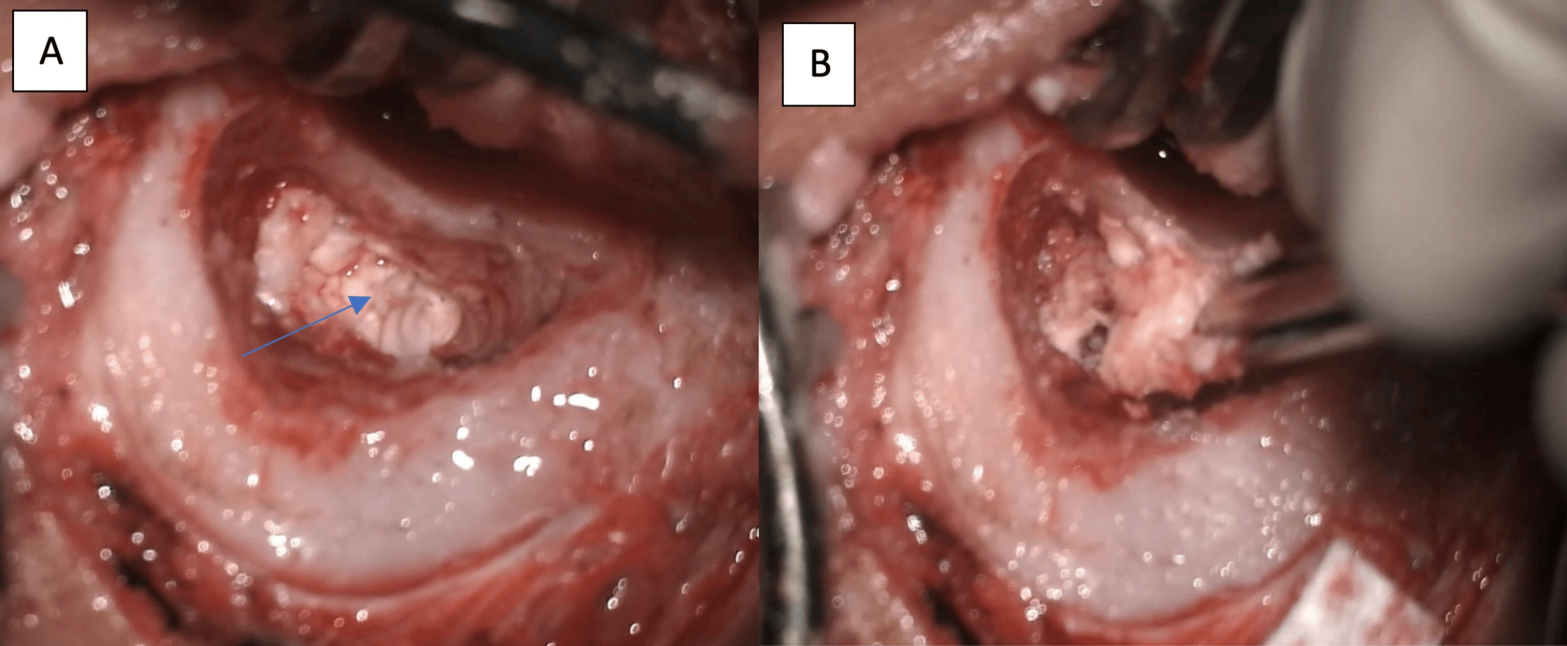
(A, B) Surgical entry via mastoidectomy approach showing evidence of mastoid involvement by cholesteatoma

Surgical intervention was carried out using a combined approach (endoscopic and microscopic). We started with the right ear, where the cholesteatoma was more extensive and the hearing loss was more significant. An initial endoscopic transcanal technique was employed, involving a standard incision and elevation of the tympanomeatal flap (Figure 5), which exposed an intact congenital cholesteatoma sac located within the middle ear (black arrow). Although the endoscopic route provided excellent visualization of the anterior and medial compartments, complete removal of the lesion was not feasible through this approach alone. Consequently, a canal wall-up mastoidectomy was performed (Figure 6), allowing access to and excision of the residual cholesteatoma extending posteriorly into the mastoid cavity. This combined technique ensured complete eradication of the lesion while preserving anatomical structures as much as possible.

The malleus and incus, which were lysed, were removed, and for the bony reconstruction, a partial prosthesis was implanted since the stapes superstructure was preserved.

**Figure 7:**
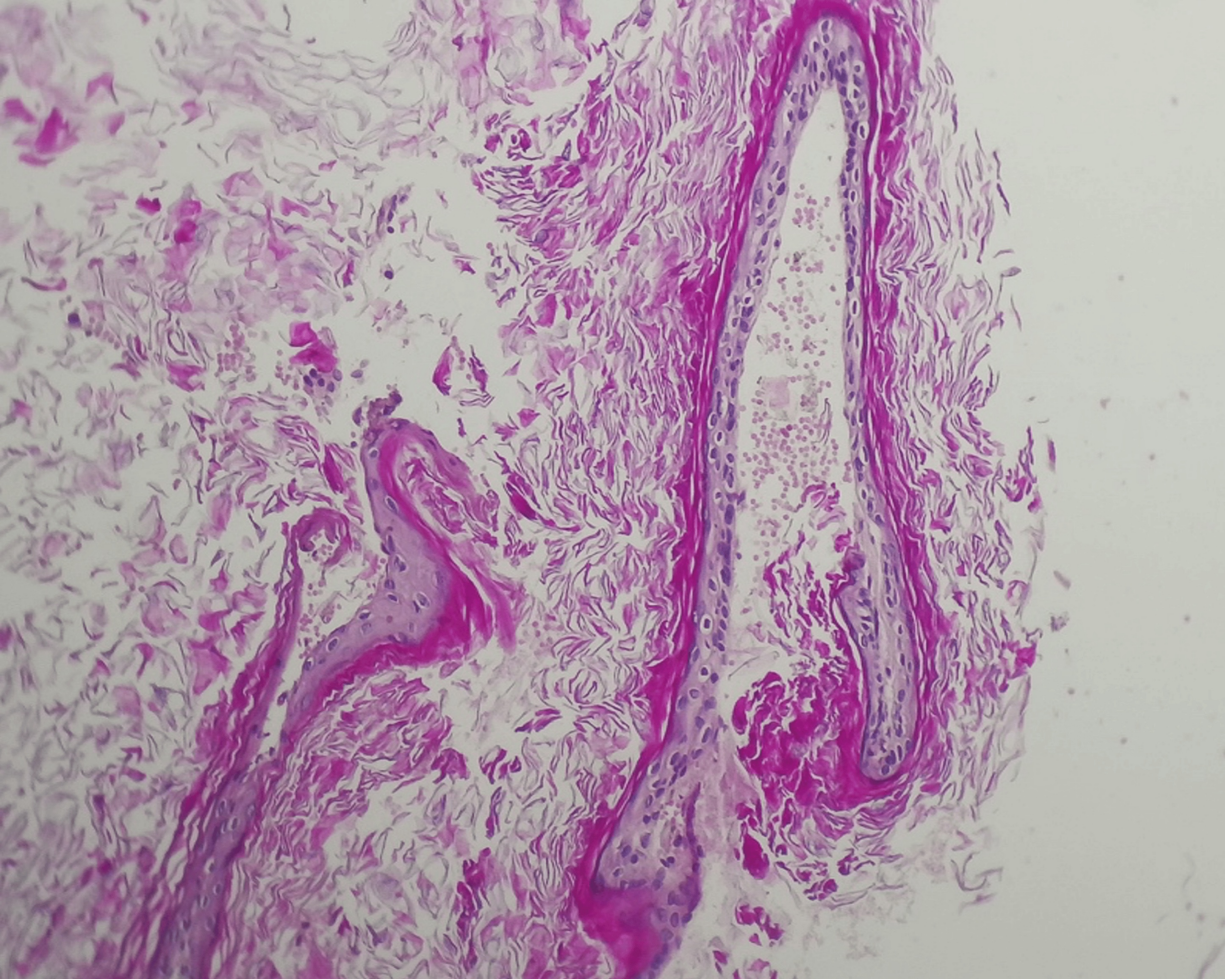
Photomicrograph of the cholesteatoma showing stratified squamous epithelial lining with prominent keratinization (H&E, ×200).

Histopathological analysis supported the diagnosis of a congenital cholesteatoma. The photomicrograph shows a cystic lesion lined by stratified squamous epithelium with prominent keratinization, without any associated inflammatory changes or granulation tissue (Figure 7). These features are characteristic of congenital cholesteatoma, which typically presents as an epithelial inclusion cyst containing lamellated keratin and lacking signs of prior infection or tympanic membrane perforation.

## Discussion

The most widely accepted etiopathogenetic theory is that proposed by Leslie Michaels. According to this theory, congenital cholesteatoma results from the persistence of an embryonic epidermoid cell rest located in the anterosuperior (AS) quadrant of the tympanic cavity. This epithelial rest, which originates from ectodermal tissue during fetal development, is normally expected to regress by the 33rd week of gestation. Failure of this involution leads to the formation of a keratinizing epithelial cyst, which gradually expands within the middle ear.

This finding accounts for the typical initial location of congenital cholesteatomas at the level of the promontory [5,6]. Several alternative hypotheses have been proposed, including the migration of embryonic epidermal cells through Gerlach’s annular ridge, amniotic contamination by epithelial cells, and metaplasia of the middle ear mucosa.

Congenital cholesteatoma should be suspected in patients with otologic symptoms, such as hearing loss or ear fullness, especially when there is no history of ear infections or surgery. Unlike acquired cholesteatoma, it arises without prior ear disease, often in children, and this was the case of our patient [7].

The diagnosis of congenital cholesteatoma is typically suggested by otoscopic examination, which reveals a whitish mass behind an intact tympanic membrane as in our case. Further imaging, such as a temporal bone CT scan or diffusion-weighted MRI, may be performed to confirm the diagnosis and evaluate the extent of the lesion. Ultimately, a definitive diagnosis is established intraoperatively [8].

Congenital cholesteatomas most commonly originate in the AS quadrant and progressively extend toward the posterosuperior quadrant, leading to ossicular erosion and eventual mastoid involvement [9].

Imaging, particularly high-resolution CT of the temporal bones, plays a crucial role in confirming the diagnosis by demonstrating a retrotympanic, hypodense, spherical lesion. CT imaging also enables precise evaluation of locoregional extension and detailed assessment of associated ossicular involvement. A systematic assessment of the contralateral ear is recommended to detect bilateral cholesteatoma or associated congenital anomalies [10]. According to Levenson and Parisier, an ossicular malformation is associated with congenital cholesteatoma in approximately 15% of cases [11].

In our patient, the diagnosis was made by otoscopic examination, with confirmation and assessment of its extent by CT scan, and was ultimately confirmed by histological analysis of the surgical specimen.

In the setting of bilateral cholesteatomatous disease, complete eradication of the cholesteatoma while preserving auditory function is of paramount importance.

Management of bilateral cholesteatoma presents a unique surgical challenge, as the disease affects both ears and thus poses a significant risk to bilateral hearing. Achieving a delicate balance between radical removal of the cholesteatomatous tissue and preservation of middle ear structures essential for hearing is a key objective of treatment. Any compromise in surgical strategy that favors preservation over complete removal can increase the risk of residual or recurrent disease.

Given the aggressive and infiltrative nature of cholesteatoma, particularly in bilateral cases, there is a significantly elevated risk of both recurrence and residual disease even after technically successful surgery. For this reason, rigorous and long-term postoperative surveillance is crucial. This includes regular otoscopic evaluations, imaging when necessary (such as diffusion-weighted MRI), and possibly a planned second-look procedure to confirm disease clearance.

Ossiculoplasty, the reconstruction of the ossicular chain, plays a central role in restoring hearing function after cholesteatoma removal. However, due to the risk of recurrence and ongoing inflammation, ossiculoplasty may either be performed during the initial surgery (primary ossiculoplasty) or delayed until a second-look surgery (staged ossiculoplasty). Delaying the procedure allows the surgeon to ensure a disease-free environment, which improves the chances of long-term success and prosthesis stability.

Studies have reported recurrence or residual cholesteatoma rates ranging from 19.7% to 48%, depending on factors such as surgical technique, the extent of the disease, and whether ossiculoplasty was staged. These figures underscore the importance of individualized treatment planning and close follow-up to optimize both disease control and auditory outcomes [12].

## Conclusions

Congenital cholesteatoma is a rare otologic condition, with bilateral involvement being exceedingly uncommon. It should be considered in the differential diagnosis of any pediatric patient presenting with otologic symptoms, particularly conductive hearing loss, in the absence of a history of otorrhea or previous otologic surgery. Diagnosis can often be suggested through a simple otoscopic examination, while imaging is essential to confirm the diagnosis, evaluate the extent of locoregional involvement, and detect any associated anatomical malformations. The treatment is based on complete surgical excision of the cholesteatoma, with a parallel aim of preserving hearing. Postoperative follow-up must be close and rigorous in order to identify any recurrence or residual disease at an early stage.
